# Transcriptomic analysis reveals distinct resistant response by physcion and chrysophanol against cucumber powdery mildew

**DOI:** 10.7717/peerj.1991

**Published:** 2016-05-17

**Authors:** Yanping Li, Shilin Tian, Xiaojun Yang, Xin Wang, Yuhai Guo, Hanwen Ni

**Affiliations:** 1 College of Agronomy and Biotechnology, China Agricultural University, Beijing, China; 2 Novogene Bioinformatics Institute, Beijing, China; 3 Institute of Animal Genetics and Breeding, College of Animal Science and Technology, Sichuan Agricultural University, Ya’an Sichuan, China

**Keywords:** Cucumber, Transcriptomic, Gene expression, RNA-seq

## Abstract

Physcion and chrysophanol induce defense responses against powdery mildew in cucumbers. The combination of these two compounds has synergistic interaction against the disease. We performed RNA-seq on cucumber leaf samples treated with physcion and chrysophanol alone and with their combination. We generated 17.6 Gb of high-quality sequencing data (∼2 Gb per sample) and catalogued the expressions profiles of 12,293 annotated cucumber genes in each sample. We identified numerous differentially expressed genes that exhibited distinct expression patterns among the three treatments. The gene expression patterns of the Chr and Phy treatments were more similar to each other than to the Phy × Chr treatment. The Phy × Chr treatment induced the highest number of differentially expressed genes. This dramatic transcriptional change after Phy × Chr treatment leaves reflects that physcion combined with chrysophanol treatment was most closely associated with induction of disease resistance. The analysis showed that the combination treatment caused expression changes of numerous defense-related genes. These genes have known or potential roles in structural, chemical and signaling defense responses and were enriched in functional gene categories potentially responsible for cucumber resistance. These results clearly demonstrated that disease resistance in cucumber leaves was significantly influenced by the combined physcion and chrysophanol treatment. Thus, physcion and chrysophanol are appealing candidates for further investigation of the gene expression and associated regulatory mechanisms related to the defense response.

## Introduction

Cucumber powdery mildew caused by *Sphaerotheca fuliginea* (Schlechtend.) Pollacci, is a widespread disease in greenhouse and field crops, causing large yield losses (Askary, Benhamou & Brodeur, 1997; Liu & Shao, 1994; Reuveni, Agapov & Reuveni, 1996). The disease has been controlled effectively by synthetic fungicides and resistance cultivars (Cohen, 1993). However, intensive and long-term application of synthetic fungicides has caused environmental pollution, residual toxicity and increased resistance to the fungicides’ active ingredients (Yang et al., 2007).

Some botanical fungicides activate plants’ natural defense systems and are known collectively as plant activators. Although plant activators and their metabolites have no direct fungicidal activity, they stimulate the immune system of plants and induce plants to display broad-spectrum, persistent and hysteresis acquired disease resistance. Therefore, plant activators would decrease the above-mentioned negative effects of synthetic agents. In this respect, plant activators are effective, selective, biodegradable, and less toxic to the environment. The development of new botanical fungicides with high efficacy, low toxicity and low residues would be beneficial to control plant disease (Bowers & Locke, 2000; Choi et al., 2004; Daayf & Bélanger, 1997; Daayf, Schmitt & Belanger, 1995; Tang, Wang & Zhang, 2004; Wurms et al., 1999; Yu et al., 2004; Zhang et al., 2008). Physcion and chrysophanol are natural anthraquinone derivatives found in plant families (Fig. S3). They are the major active ingredients in traditional herbal medicines that are widely used in clinics (Xu, Huang & Yang, 2004). Physcion and chrysophanol have numerous biological activities, including anti-inflammatory, antitumor, antioxidant, antifeedant and antimicrobial (Barnard et al., 1992; Boik, 1995; Chang et al., 1996; Evans, 1996; Huang et al., 1992; Huang, Chu & Chao, 1991; Singh et al., 1992; Sun et al., 2000; Trial & Dimond, 1979; Yen, Duh & Chuang, 2000; Zhan et al., 2000), which could affect the vasomotor system, immune system and metabolic processes.

Physcion and chrysophanol also have activity against phytopathogens, such as barley powdery mildew (*Blumeria. graminis f. sp. Hordei*), cucumber powdery mildew (*S. fuliginea*) (Choi et al., 2004), rice sheath blight (*Rhizoctonia solani* Kuhn), grey mold (*Botrytis cinerea* Pers ex Pers) and cucumber downy mildew (*Pseudoperonospora cubensis* de Bary) (Yu et al., 2006).

Previous studies showed that physcion inhibited conidial germination of *B. graminis* by nearly 100% if applied on barley leaves before inoculation (Yang et al., 2008). Resistance risk evaluation showed that this chemical had a low risk in powdery mildew and downy mildew populations (Yang et al., 2008). The bioactivity of physcion against the barley powdery mildew pathogen was better than that of the other anthraquinones in pot tests. The inhibitory effect of chrysophanol on spore germination and mycelia growth of *S. fuliginea* was reported (Tang et al., 2002). Chrysophanol has both protective and curative activity against cucumber powdery mildews, and has high activity against *S. fuliginea*, including reducing the spore germination rate, depressing the growth of mycelia and reproduction of new conidia (Ren et al., 2008; Ren et al., 2009; Ren, Fan & Cao, 2012). Previous studies also showed that there was a synergistic interaction between physcion and chrysophanol against powdery mildew pathogens (Ma et al., 2010; Yang, 2007; Yang et al., 2007).

However, the mechanism of the synergistic interaction between physcion and chrysophanol against powdery mildew pathogens has not been determined. To explore the synergistic mechanism of physcion and chrysophanol action against the pathogen *S. fuliginea* at the transcriptional level, we used the whole RNA-seq to determine the transcriptional differences in cucumber leaves treated with physcion and chrysophanol alone and their combination in comparison with solvent-treated controls.

## Materials and Methods

### Plant materials and growth conditions

Seeds of cucumber cultivar Changchunmici, which is highly susceptible to cucumber powdery mildew, were washed three times with sterile water and transferred to a water bath pot at 25 °C to soak for 4–6 h. The treated seeds were placed in Petri dishes with filter paper at the bottom and two layers of gauze above and incubated at 25 °C for 24 h. When the buds grew to about 5 mm long, they were sown in plastic pots containing sterilized soil (200 mm in diameter, 10 plants per pot) in a growth chamber at 25 °C. When the fourth leaves were fully expanded, the plants were ready for compound treatment and conidium inoculation.

### Inoculum of *S. fuliginea*

Isolates of *S. fuliginea* were provided by the Plant Pathology Lab of the Institute of Plant Protection and Soil Science, Hubei Academy of Agricultural Sciences, China. Cucumber powdery mildew was reproduced in a growth chamber at 25 °C and 70% relative humidity with a 16-hour light and 8-hour dark photoperiod. Once adequate amounts of spores had been produced for the experiment, an inoculation suspension of *S. fuliginea* (about 2 × 10^5^ spores mL^−1^) was prepared.

### Compound treatment and sampling

Physcion (98%) and chrysophanol (98%) were obtained from the National Institute for the Control of Pharmaceutical and Biological Products, Beijing, China. Stock solutions were prepared by dissolving physcion (10 mg) and chrysophanol (10 mg) in 5 mL of dimethylsulfoxide (DMSO), separately, which were stored at 4 °C in the dark before application.

To explore the synergistic mechanism of physcion and chrysophanol against the pathogen *S. fuliginea* in cucumber, physcion and chrysophanol alone and their combination were applied to the cucumber plants before inoculation at the concentration of 10 mg/L. The surfactant Tween 20 was added to the final dilutions at the concentration of 0.25 g/L, respectively, and solvent-treated (0.5% DMSO and 0.25% Tween 20) plants were used as controls. Tested plants were inoculated by spraying *S. fuliginea* spore suspension 8 h after treatment. After inoculation, the plants were incubated at 25 °C and 70% relative humidity until sampling. The fourth leaves of the cucumber plants were collected at 3 days post-inoculation (dpi) when solvent-treated control plants displayed disease symptoms. All samples were immediately frozen in liquid nitrogen and stored at −80 °C until further use.

### Total RNA isolation, library preparation and sequencing

Total RNA was isolated from each sample using the TRIzol reagent (Life Technologies, Beijing, China), according to the manufacturer’s protocols. The concentration of each RNA sample was determined using a Qubit2.0 (Life Technologies, CA, USA) and RNA integrity was confirmed using a 2100 Bioanalyzer (Agilent Technologies Inc., Santa Clara, CA, USA) with a minimum RNA integrity number (RIN) value of 8.0 and with 250 ng of total RNA per sample. Three micrograms of RNA per sample was used as input material to prepare the libraries following the standard procedures of TruSeq RNA Sample Prep Kit v2 (Illumina, San Diego, CA, USA). In brief, the poly (A) mRNA was extracted from total RNA using poly-T-attached magnetic beads, further fragmented, and used as templates for cDNA generation. First strand cDNA was synthesized using random hexamer primers and reverse transcriptase. Second strand cDNA was synthesized based on the first strand with dNTP, buffer solution, DNA polymerase I and RNase H. Double-stranded cDNA was then purified using AMPure XP beads, and the second strands (containing uridines) were degraded using the USER Enzyme. Purified double-stranded cDNA was then end-repaired, a poly(A) added, and ligated to paired-end Illumina sequencing adaptors. cDNA fragments of about 320 bp were size-selected using AMPure XP beads and amplified using PCR. The high-quality libraries were sequenced on an Illumina HiSeq™ 2,000 platform and 100-bp paired-end (PE) reads were generated using the TruSeq SBS Kit v3-HS (Illumina, Inc.). Bases were called using the Illumina CASAVA software.

The transcriptome sequencing data have been deposited in the Gene Expression Omnibus under accession code: GSE72034.

### Read mapping and data processing

Initially, low-quality reads (phred ≤20) and adaptor sequences were filtered out, and the Q20, Q30 and GC content of the clean data were calculated. All the subsequent analyses were based on the high-quality, clean data. The reference genome and gene model annotation files were downloaded from the cucumber genome website (http://cucumber.genomics. org.cn/). An index of the reference genome was built using Bowtie v2.0.6 (Broad Institute, Cambridge, MA, USA) and paired-end clean reads were aligned to the reference genome using TopHat v2.0.9 (Broad Institute) with the default parameters. HTSeq v0.5.3 (EMBL, Heidelberg, Germany) was used to count the read numbers mapped to each gene. The reads per kilobase of transcript per million mapped reads (RPKM) of each gene were calculated based on the length of the gene and read count mapped to it. We carried out read alignment and expression quantification separately for each sample. Only genes with fragments per kilobase of transcript per million mapped reads (FPKM) values larger than four and that exhibited low variation across three biological replicates (coefficient of variation ≤30%) were considered reliable and were used in subsequent analyses.

### Identification of differentially expressed genes (DEGs)

Identification of DEGs among the four treatments (i.e., physcion alone, chrysophanol alone, the combination treatment and the solvent-treated control sample) was performed using the DESeq R package v1.12.0, which determines DEGs using a model based on the negative binomial distribution. The resulting *P*-values were adjusted using Benjamini and Hochberg’s approach to control the false discovery rate (FDR). The significance of the gene expression difference was indicated by an adjusted *P*-value <0.05, as determined by DESeq.

### Functional enrichment analyses for DEGs

Gene functional enrichment analysis of DEGs was implemented using the GOSeq R package, in which gene length bias was corrected. Gene ontology (GO) terms involving cellular component (CC), molecular function (MF), and biological process (BP), as well as the Kyoto Encyclopedia of Genes and Genomes (KEGG) pathways and InterPro database, were considered significantly enriched by DEGs when their Benjamini adjusted *P*-values were ≤0.05.

## Results and Discussion

### Sequencing data summary

We constructed 10 RNA-seq libraries from cucumber leaves that had been treated by chrysophanol (termed Chr-1 and -2), physcion (termed Phy-1, -2 and -3), combination of physcion and chrysophanol (termed Phy × Chr-1, -2 and -3) or the solvent-treated control (termed Con-1 and -2). Each RNA library was sequenced individually, which generated ∼15.32–19.45 M reads for each library. After filtering out the adaptor tags and low-quality tags, we obtained clean reads ranging from 14.89 to 18.88 M. The clean reads accounted for more than 97% of the total, which were then mapped to the cucumber genome for further gene expression analysis (Table S1 and Table S2). Among 23,907 annotated genes in the cucumber reference genome, after filtering the low expression levels genes with log2 (RPKM) ≤2 at least one sample, we quantified the expression levels of 12,293 (51.42%) genes across 10 samples. An inspection of transcript homogeneity was performed to confirm that each library had sufficiently high coverage depth and uniformity (Fig. S1).

## Global gene expression profiling analysis

The biological replicates within each treatment correlated highly with each other (Average Pearson’s *r*value = 0.92), which suggested experimental reliability and further highlighted the low variation in the transcriptomes activated by botanical fungicides in leaves (Fig. S2 and Table S3).

**Figure 1:**
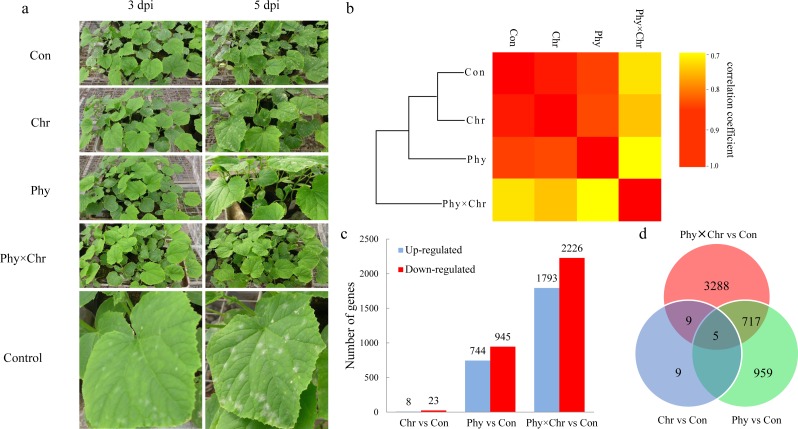
The global transcriptome similarity and differences caused by physcion and chrysophanol treatment for cucumber powdery mildew. (A) Histological investigation of leaves inoculated with *S. fuliginea* at 3 dpi and 5 dpi after the four treatments. (B) Hierarchical clustering and heat map matrix of pairwise Pearson’s correlations of the expression profiles in leaves between the four treatments (solvent-treated control; chrysophanol treatment; physcion treatment; physcion and chrysophanol combination treatment) (log_2_-fold changes). (C) The up- and down-regulated DEGs. (D) A Venn diagram displaying the distribution of the DEGs.

Compared with the relatively high correlations among the Chr and Phy alone treatments, the Phy × Chr treatments exhibited lower correlation rates, indicating differences in gene expression patterns in the Phy × Chr treatments. Hierarchical clustering confirmed these findings. The samples were almost perfectly clustered by the different treatments. Among the four treatments, the solvent-treated control and the Chr treatment were closer to each other than to the Phy and Phy × Chr treatments. The Chr and Phy treatments were also closer to each other than to the Phy × Chr treatment (Fig. 1A), which indicated that the gene expression patterns of the Chr and Phy treatments were more similar to each other than to the Phy × Chr treatment. The relatively similar global gene expression profiles between the Chr and Phy treatments might indicate a similar effect on cucumber powdery mildew pathogens, while the Phy × Chr treatment might have a different effect on cucumber powdery mildew pathogens. These results are consistent with histological investigation of cucumber powdery mildew pathogens. In agreement with the transcriptome differences, there were significant differences in the pathogen symptom severity in the four groups of cucumber leaves at 3 dpi (Fig. 1A). The leaves from the solvent-treated control had the highest pathogen symptom severity at 3 dpi. The leaves from the chrysophanol alone treatment had a higher pathogen symptom severity than those from physcion alone treatment at 3 dpi. Leaves treated with physcion combined with chrysophanol had the fewest pathogens symptom severity. The chrysophanol and physcion alone groups had significant pathogen symptom severity at 3 dpi and 5 dpi, respectively, while the combined group had no pathogen symptom severity until 5 dpi. These results clearly demonstrated that disease resistance in cucumber leaves was markedly influenced by the physcion and chrysophanol combination treatment.

### Identification of DEGs

To explore the differences among the transcriptomes induced by physcion and chrysophanol, we performed separate pairwise comparisons between the three treatments against the solvent-treated control sample. Consequently, we identified 31 DEGs (log_2_ fold-change >2.0 and a *P* < 0.05) between the Chr treatment and solvent-treated control samples, which was less than that of Phy treatment leaves (1,689 DEGs) and Phy × Chr treatment leaves (4,019 DEGs). Nine hundred and fifty-nine genes were specifically regulated after physcion treatment and 3,288 genes were specifically regulated after the combined. Among three treatments, the Phy × Chr treatment induced the highest number of DEGs. This dramatic transcriptional change induced by the Phy × Chr treatment leaves reflects that the combined treatment was most closely associated with induction of disease resistance (Fig. 1B and Table S4). Venn diagram analysis (Fig. 1C and Table S5) showed a unique set of genes in Phy × Chr treatment leaves, which suggests that the combined treatment had a significant effect on the transcription of a subset of genes.

### Functional annotation of DEGs

**Figure 2:**
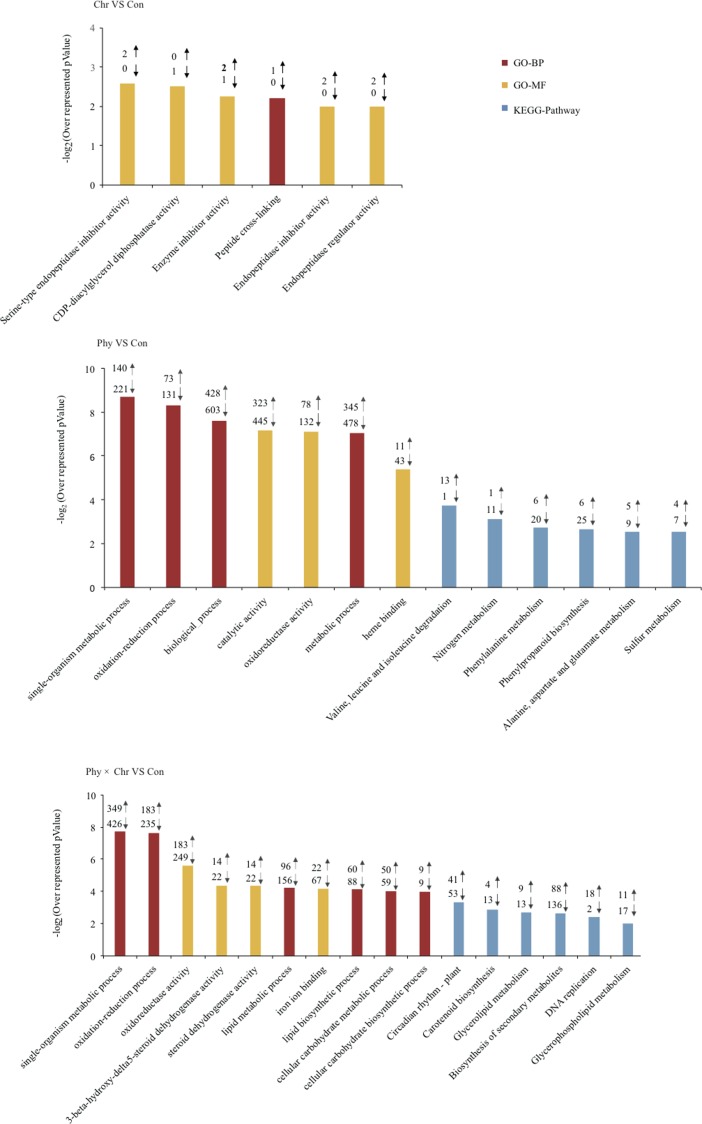
Top 10 Go (gene ontology) and pathway categories enriched for up- (up arrows) and down-regulated (down arrows) genes differentially expressed in cucumber leaves activated by chrysophanol and/or physcion. The *p* value, indicating the significance of the comparison, was calculated using a Benjamini-corrected modified Fisher’s exact test. BP, biological process; MF, molecular function.

Genes showing altered expression in each comparison against the solvent-treated control were analyzed using the DAVID tool to examine whether these DEGs were enriched for specific biological processes (Huang et al., 2007). As expected, numerous DEGs were significantly over-represented in the categories related to the defense response. Typically, DEGs induced by the Phy × Chr treatment were significantly enriched for the cell wall-related categories, including the plant-type cell wall (six genes, *P* = 0.0037), chloroplast (six genes, 0.022) and pigment catabolic process (10 genes, *P* = 0.018) (Fig. 2 and Table S6). This finding may reflect the essential roles of cell wall-related categories as structural defenses in response to pathogens. Characteristically, the cell wall is a major line of defense against fungal and bacterial pathogens and provides an excellent structural barrier.

In addition, DEGs induced by the Phy × Chr treatment were also enriched in the categories of lipid metabolic process (156 genes, *P* = 2.0 × 10^−6^), lipid biosynthetic process (88 genes, *P* = 1.1 × 10^−4^), oxidation–reduction process (235 genes, *P* = 1.7 × 10^−5^), oxidoreductase activity (249 genes, *P* = 2.5 × 10^−5^) and oxidoreductase activity of acting on paired donors (81 genes, *P* = 2.5 × 10^−6^); and in subcategories of ‘binding’, such as iron ion binding (67 genes, *P* = 1.1 × 10^−8^), heme binding (61 genes, *P* = 2 × 10^−4^), tetrapyrrole binding (61 genes, *P* = 5.8 × 10^−4^), transition metal ion binding (208 genes, *P* = 0.02) and heat shock protein binding (16 genes, *P* = 0.039) (Fig. 2 and Table S6). DEGs were associated with lipid metabolism corresponding to cutin, suberine and wax biosynthesis, which is a well-known chemical defense pathway in response to pathogens. Cutin and suberine are the polymer matrices for lipophilic cell wall barriers. Oxidoreductases are involved in the biosynthesis of these polymers (Pollard et al., 2008) and could catalyze an oxidation–reduction (redox) reaction in response to pathogens; these results are consistent with previous studies (Freeman, 2008; Rahfeld et al., 2014). DEGs in subcategories related to ‘binding’ may play an important role in catalysis and substrate binding in response to pathogens. All the above-mentioned categories enriched by DEGs belong to chemical defenses, which incorporates a wide variety of structural defenses that are rapidly activated when the cell detects the presence of potential pathogens.

In addition, DEGs induced by Phy × Chr treatment were also enriched in the categories associated with signaling, such as response to abiotic stimulus (12 genes, *P* = 9.3 × 10^−4^) and response to chemical stimulus (23 genes, *P* = 0.04), phosphorylation (130 genes, *P* = 1.9 × 10^−3^) and protein phosphorylation (117 genes, *P* = 4.7 × 10^−3^). Enrichment of response to stimulus component was consistent with signaling-related pathogen perception and induction of the resistance response (Michele, Cesare & Broggini, 2013). Phosphorylation plays critical roles in plant disease resistance by regulating multiple defense responses (Xiangzong et al., 2013) (Fig. 2 and Table S6).

The KEGG pathway enrichment analysis indicated that the DEGs are involved in resistance-related metabolic pathways. The major pathways regulated by chrysophanol and physcion were revealed as biosynthesis of secondary metabolism, phenylpropanoid biosynthesis, glyceropospholid metabolism, cutin, suberine and wax biosynthesis (Fig. 2 and Table S7).

### DEGs involved in structural, chemical, and signaling defense responses

Resistance in many plant–pathogen interactions is accompanied by the rapid deployment of a multicomponent defense response comprising structural, chemical, and signaling moieties.

**Figure 3:**
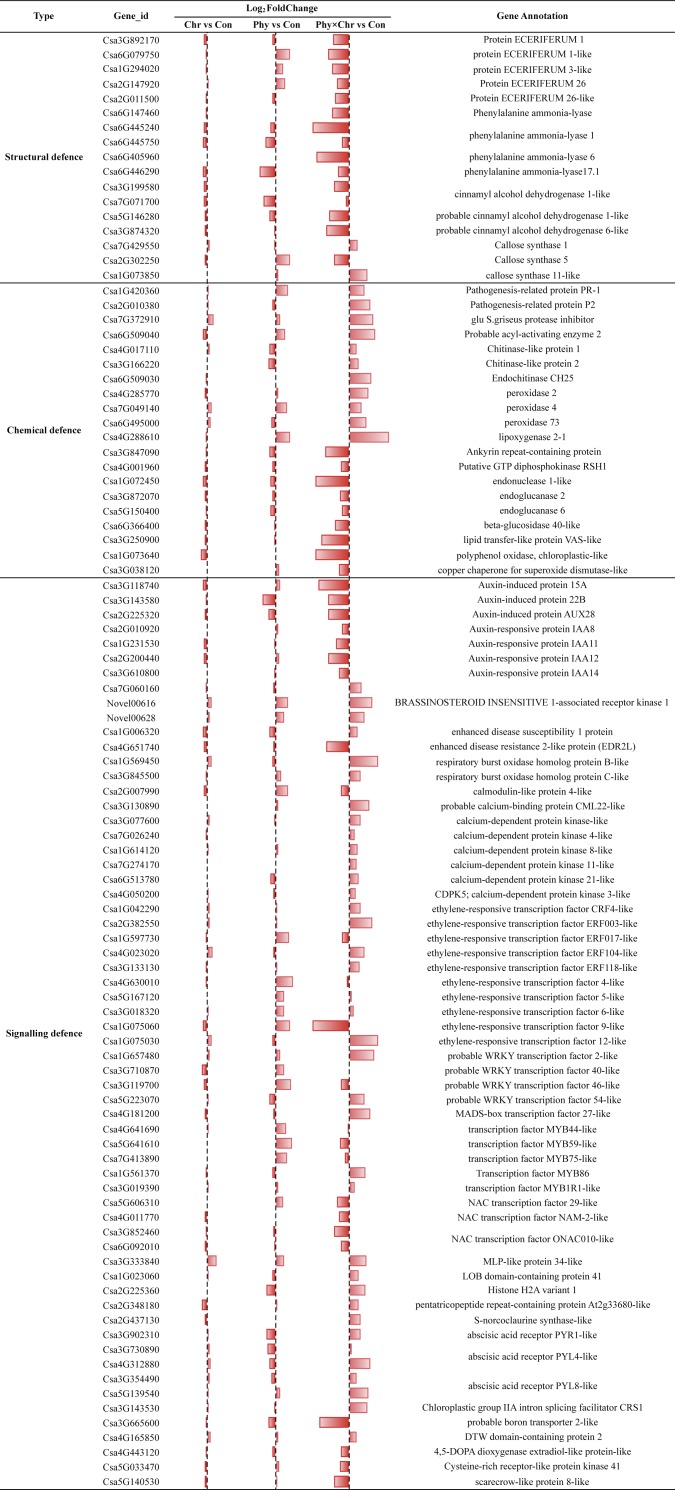
Comparison of the proportions of defense-related genes in Chr1, Phy1 and Phy × Chr vs. Con1. A bar left of the line means that the gene is down-regulated, while a bar right of the line means up-regulated.

Our result showed that comparable proportions of DEGs in Chr, Phy and Phy × Chr were over-represented in the candidate gene set, including 17, 20 and 62 genes involved in structural, chemical and signaling-related defense reactions, respectively (Table S8). Nonetheless, the specific gene content within this set differs significantly in Chr1, Phy1 and Phy × Chr (Fig. 3 and Table S8).

For the structural defense, the cell wall is a major line of defense against fungal pathogens. Among the cell wall precursors, those of wax and lignin play a role in structural defense. We compared the transcriptome profiles of the solvent-treated Control, Chr, Phy, and Phy × Chr treatments and found that Chr treatment alone had little effect on the induction of structural defense-related genes. Phy treatment alone induced the transcription of several structural defense-related genes, while Phy × Chr treatment induced the transcription of a subset of genes involved in structural defense, such as wax precursor protein ECERIFERUM (Csa3G892170, Csa6G079750, Csa1G294020, Csa2G147920, Csa2G011500) and downregulated lignin precursors-associate genes such as phenylalanine ammonia-lyase (Csa6G147460, Csa6G405960, Csa6G445240) and cinnamyl alcohol dehydrogenase (Csa3G199580, Csa3G874320, Csa5G146280, Csa7G071700). Thus, a reduced production of coumaryl-, caffeyl-, coniferyl-, hydroxyconiferyl- and sinepsyl-alcohols for phenylpropanoid alcohols would be expected.

Cellulose synthase is another essential enzyme for the formation of the cell wall, which is the primary interface for plant–pathogen interaction (Dodds & Rathjen, 2010; Paula, Roger & Sheng Yang, 2003; Rosli et al., 2013) Our analysis found that the callose synthase gene (Csa7G429550, Csa1G073850) was upregulated in Phy × Chr-treated leaves. Similarly, lignin precursors phenylalanine ammonia-lyase genes (Csa6G445750, Csa6G446290) were downregulated and the callose synthase gene (Csa2G302250) was upregulated in Phy treatment leaves, which provides a possible explanation for Phy treatment alone inducing resistance to powdery mildew. The results showing that the plants subjected to the combined treatment had the highest resistance to powdery mildew pathogens were consistent with previous observations (Yang et al., 2007) (Fig. 3 and Table S8).

In chemical defense, plant secondary metabolites are not directly involved in growth or reproduction, but act as chemical barriers. They were linked to plant activator-induced resistance in systemic-acquired resistance (SAR) (Soylu, Baysal & Soylu, 2003). In the present study, genes encoding pathogenesis-related protein (PR, Csa1G420360, Csa2G010380), glu S. griseus protease inhibitor-like (Pin, Csa7G372910), chitinase (Csa4G017110, Csa6G509030, Csa6G509040, Csa3G166220), peroxidase (PX, Csa7G049140, Csa6G495000, Csa4G285770) and lipoxygenase (LOX, Csa4G288610) were upregulated, while those encoding endonuclease (Csa1G072450, Csa3G847090, Csa4G001960), endoglucanase (Csa3G872070, Csa5G150400, Csa6G366400), lipid transfer-like protein VAS-like (LTP, Csa3G250900), polyphenol oxidase (Csa1G073640) and superoxide dismutase (Csa3G038120) were downregulated in Phy × Chr-treated leaves. This is also consistent with a previous report (Blein et al., 2002; In Sun & Byung Kook, 2010; Rancé, Fournier & Esquerré-Tugayé, 1998). The expression levels of PR and Pin genes increased following inoculation in plant activator-treated plants. Chitinases catalyze the degradation of chitin, a polymer with a backbone similar to cellulose that is present in the cell walls of true fungi (Freeman, 2008). The biochemical functions of PXs are associated with lignin and suberin biosynthesis (Quiroga et al., 2000) and with the regulation of reactive oxygen species (ROS) (Kawano, 2003). Silencing a plant PX gene resulted in increased plant pathogen susceptibility (Hyong Woo et al., 2007) and its overexpression enhanced plant resistance (Choi & Hwang, 2011). The effect of PXs on S. *fuliginea* growth may be linked to strengthening of the cell wall and the consequent reduction of the nutrient availability necessary for fungal growth (Timothy & Schumann, 2010). LOX proteins are involved in the first step of the jasmonate biosynthesis pathway (Wasternack & Kombrink, 2010). The increased expression of the LOX proteins may inhibit fungal growth by producing fungal inhibitor oxylipin substances (e.g., hexanal and colnelenic acid) or by their own antimicrobial activities (Helena & Mario, 2002; Vaughn & Gardner, 1993). Glucanases catalyze the degradation of (*Triticum aestivum*) glycosidic linkages in glucans, a class of polymers similar to cellulose that are present in the cell walls of many oomycetes. Lipid transfer proteins (LTPs) have been linked to antifungal activity through different possible paths upon pathogen attack and to a potential inhibition of germination and fungal growth *in vitro* (Blein et al., 2002; Kirubakaran et al., 2008). Polyphenol oxidase and superoxide dismutase might play a crucial role in the protection of the plant cell from oxidative damage at the sites of enhanced ROS generation (Soylu, Baysal & Soylu, 2003) (Fig. 3 and Table S8).

In signaling defense, plants have evolved intricate mechanisms to perceive external signals, thereby enabling an optional response to biotic and abiotic stimuli. A number of signaling pathways with roles in regulating the response to pathogens have been defined (Yang et al., 2015).

The plant hormones such as jasmonic acid, salicylic acid and ethylene are not only important signaling molecules, but also play a critical role in the regulation of plant immune responses. In this study, Chr alone and Phy alone and their combination all repressed the transcription of nearly all of the genes in the auxin-mediated signaling pathways. These genes encode auxin-induced protein (Csa2G225320, Csa3G143580, Csa3G118740) and auxin-responsive protein IAAs (Csa2G200440, Csa3G610800, Csa2G010920, Csa1G231530). This could be related to the cessation of cell enlargement and cell division hormone response in the Chr alone and Phy alone or combination-treated leaves. Phy × Chr treatment up-regulated the expression of the gene encoding the BRASSINOSTEROID INSENSITIVE 1-associated receptor kinase 1-like (Csa7G060160, Novel00616, Novel00628), which is involved in brassinosteroids (BRs) metabolism and signaling. BRs have long been seen as mainly positive players in plant immunity. Recent findings in both dicots and rice suggested a more complex situation, with positive, negative and neutral effects of BRs being reported, which are seemingly independent of either the plant species or type of pathogen involved (Bruyne, Höfte & Vleesschauwer, 2014).

In addition, we identified DEGs encoding an enhanced disease susceptibility 1 (EDS1: Csa1G006320) protein and enhanced disease resistance 2-like protein (EDR2L: Csa4G651740). EDS1 was first observed in a mutant of *A. thaliana* that was susceptible to *P. parasitica*. EDS1-silencing increased disease resistance in *Arabidopsis* (Parker et al., 1996). The EDS1 gene is necessary for the functionality and signal transduction of other resistance genes in *Arabidopsis* plants (Aarts et al., 1998; Kazan & Manners, 2008). In our experiment, without considering the FDR *P*-value correction, the EDS1 gene was downregulated in both Chr and Phy-treated leaves, which potentially provides an additional explanation for the resistance to powdery mildew induced by Chr alone and Phy alone treatments. However, the mechanism by which EDS1 affects the physcion- and chrysophanol- induced resistance against cucumber powdery mildew pathogens remains to be determined. EDR2L was down-regulated in Phy × Chr-treated plants, which was consistent with a previous study in which EDR2 was observed to negatively regulate salicylic acid–based defenses and cell death during powdery mildew infection of *Arabidopsis thaliana* (Vorwerk et al., 2007).

As secondary signaling molecules, calcium and ROS are crucial for the development of plant defense against abiotic and biotic stimuli. ROS signaling is integrated with calcium signaling in plants. Here, we found that several important genes involved in cellular redox homeostasis were upregulated in Phy × Chr-treated plants, such as those encoding respiratory burst oxidase homolog protein B-like (Csa1G569450) and respiratory burst oxidase homolog protein C-like (Csa3G845500), which suggested that they are critical for the defense against powdery mildew pathogens after Phy × Chr treatment. Several important genes involved in calcium signaling were upregulated in Phy × Chr-treated plants, including those encoding calcium-dependent protein kinase-like (CDPK, Csa1G614120, Csa3G077600, Csa6G513780, Csa7G026240, Csa7G274170, Csa4G050200) and probable calcium-binding protein CML22-like (Csa3G130890). Compared with Chr treatment alone or the combination treatment, the transcription of calmodulin-like protein 4-like (Csa2G007990) was upregulated by Phy treatment alone, thus providing a possible explanation for Phy alone-induced resistance to powdery mildew.

Ethylene has been observed to induce a defense response in many plants by upregulating genes involved in ethylene production, including ACC oxidase (ACO) (Cohn & Martin, 2005; Steinhorst & Kudla, 2013). In the present study, Phy × Chr treatment activated genes encoding ethylene-responsive transcription factor-like (Csa1G075030), CRF4-like (Csa1G042290) and ERF118-like (Csa2G382550, Csa3G133130, Csa4G023020). The ethylene-responsive transcription factor-like (Csa1G075060, Csa5G167120, Csa3G018320, Csa4G630010) and ERF017-like (Csa1G597730) were also activated in Phy-treated plants, which provides another explanation for Phy treatment alone causing resistance to powdery mildew.

Transcription factors (TFs) including NAC, WRKY, MYB, AP2, bHLH, C2H2-like zinc finger, HSF and bZIP play central roles in plant abiotic and biotic stress responses by regulating downstream genes via specific binding to cis-acting elements in the promoters of target genes. Phy × Chr treatment induced a significant increase in the expressions of certain transcription factor genes, including those encoding the probable WRKY transcription factor 2-like (Csa1G657480), WRKY34 (Csa5G223070), MADS-box transcription factor 27-like (Csa4G181200), transcription factor MYB1R1-like (Csa3G019390) and MYB86 (Csa1G561370). Phy treatment alone also induced a significant increase in the expressions of genes encoding transcription factors, such as probable WRKY transcription factor 40-like (Csa3G710870) and WRKY50 (Csa3G119700), transcription factor MYB59-like (Csa5G641610) and MYB44-like (Csa4G641690). However, Phy treatment also suppressed the transcription of NAC transcription factor ONAC010-like (Csa3G852460, Csa6G092010), NAM-2-like (Csa4G011770), and 29-like (Csa5G606310). The crosstalk between induced signaling events, including receptor-mediated signal perception, protein phosphorylation, ion fluxes, production of ROS, and the generation and regulation of secondary signaling molecules, leading to the activation of defense genes, has been well established as the mechanism of host plant defense to different pathogens (Annemart & Pieterse, 2008) (Fig. 3 and Table S8).

## Conclusions

We identified DEGs in cucumber leaves treated with chrysophanol alone, physcion alone, and with chrysophanol and physcion combined, and obtained detailed expression profiles of genes involved in the response to the powdery mildew pathogen. The Phy × Chr treatment induced the highest number of DEGs. Functional annotation of DEGs identified candidate genes involved in structural, chemical, and signaling defense responses. The expression variations of structural, chemical, and signaling defense-related genes indicated that they are coordinately regulated following powdery mildew pathogen infection after chrysophanol and physcion treatment. Numerous DEGs in the Phy × Chr group are associated with defense response-associated terms, such as cell wall-related categories, lipid metabolism, response to stimulus components and oxidoreductase activity. This dramatic transcriptional change in the Phy × Chr-treated leaves reflects the fact that the combined treatment with physcion and chrysophanol was most closely associated with induction of disease resistance. The overall findings from this study increased our understanding of the molecular effects of Phy × Chr combination treatment in the cucumber and provide useful information for further studies. These results also provide a basis for exploring the complex gene expression and associated regulatory mechanisms of plant activator-induced defense responses to pathogens.

## Supplemental Information

10.7717/peerj.1991/supp-1 Figure S1 Mean coverage distribution of genes in different treatment samples The *x*-axis represents the Distance for 5’ end of transcript (%) of sample, and the *y*-axis represents Mean coverage of sample. High means high expression amount of transcript, medium means moderate expression of transcription and low means low expression of transcripts.

10.7717/peerj.1991/supp-2 Figure S2 Pearson correlation coefficients between samples The *x*-axis represents log_10_ (FPKM + 1) of sample1, and *y*-axis represents log_10_ (FPKM + 1) of sample2. R2 means the square of Pearson correlation coefficient.

10.7717/peerj.1991/supp-3 Figure S3 The chemical structures of physcion and chrysophanol

10.7717/peerj.1991/supp-4 Table S1 Data Quality Summary after illumine sequencing

10.7717/peerj.1991/supp-5 Table S2 Summary of clean reads mapped to the cucumber genome

10.7717/peerj.1991/supp-6 Table S3 Pearson correlation coefficients between samples

10.7717/peerj.1991/supp-7 Table S4 List of differentially expression genes (DEGs) (log_2_ Ratio ≥1, false discovery rate (FDR) < 0.05)

10.7717/peerj.1991/supp-8 Table S5 The gene list of Venn diagram analysis of DEGs

10.7717/peerj.1991/supp-9 Table S6 Gene Ontology enrichment analysis list of DEGs with log_2_ fold-change >2

10.7717/peerj.1991/supp-10 Table S7 Kyoto Encyclopedia of Genes and Genomes (KEGG) pathway enrichment list of DEGs

10.7717/peerj.1991/supp-11 Table S8 Functional candidate genes related to ‘defense’ in different treatment groups

## Additional Information and Declarations

### Data Availability

The following information was supplied regarding data availability: The raw data is deposited in the Gene Expression Omnibus: GSE72034.
