# Supplementary figures and images for: Transcriptomic analysis reveals distinct resistant response by physcion and chrysophanol against cucumber powdery mildew
Source: PeerJ. 2016 May 17;4:e1991. doi: 10.7717/peerj.1991 (PMC4878370; doi:10.7717/peerj.1991)

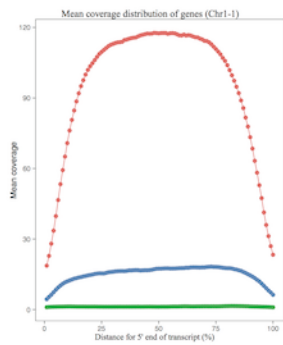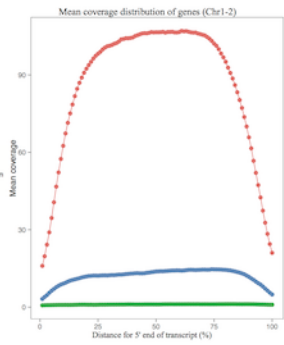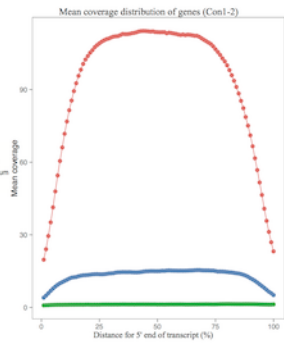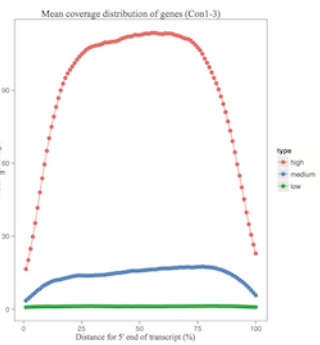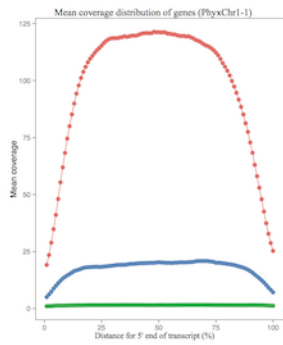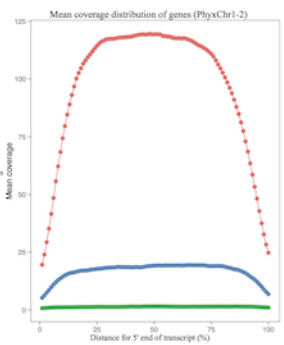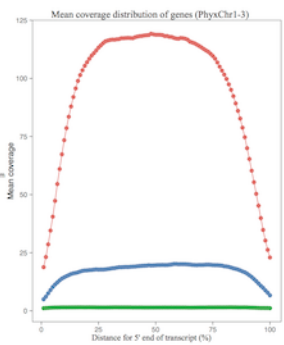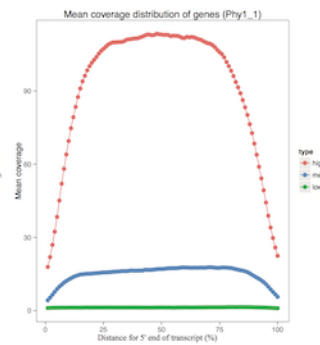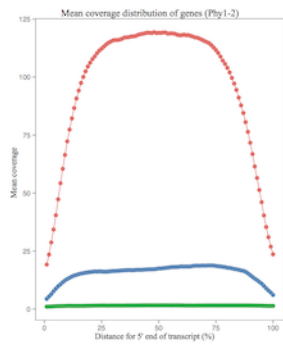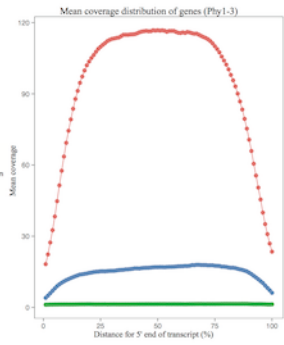

Supplement: Figure S1 — The x-axis represents the Distance for 5’ end of transcript (%) of sample, and the y-axis represents Mean coverage of sample. High means high expression amount of transcript, medium means moderate expression of transcription and low means low expression of transcripts. [file peerj-04-1991-s001.pdf]

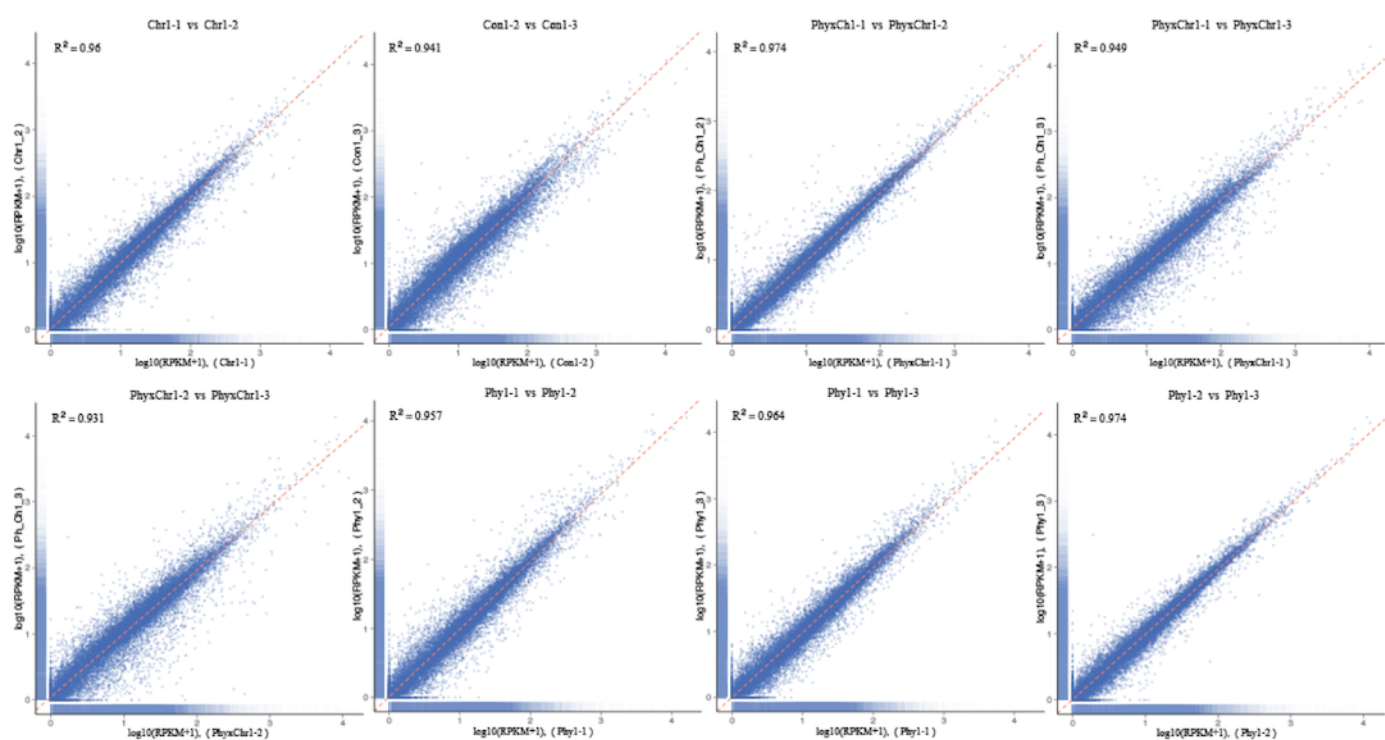

Supplement: Figure S2 — The x-axis represents log10 (FPKM + 1) of sample1, and y-axis represents log10 (FPKM + 1) of sample2. R2 means the square of Pearson correlation coefficient. [file peerj-04-1991-s002.pdf]

a

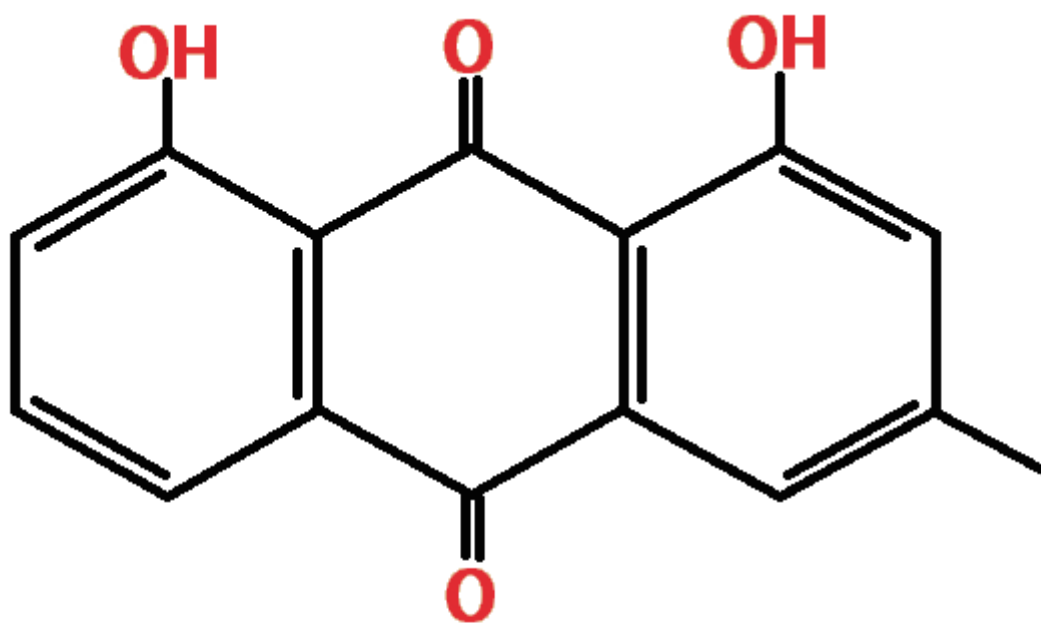

b

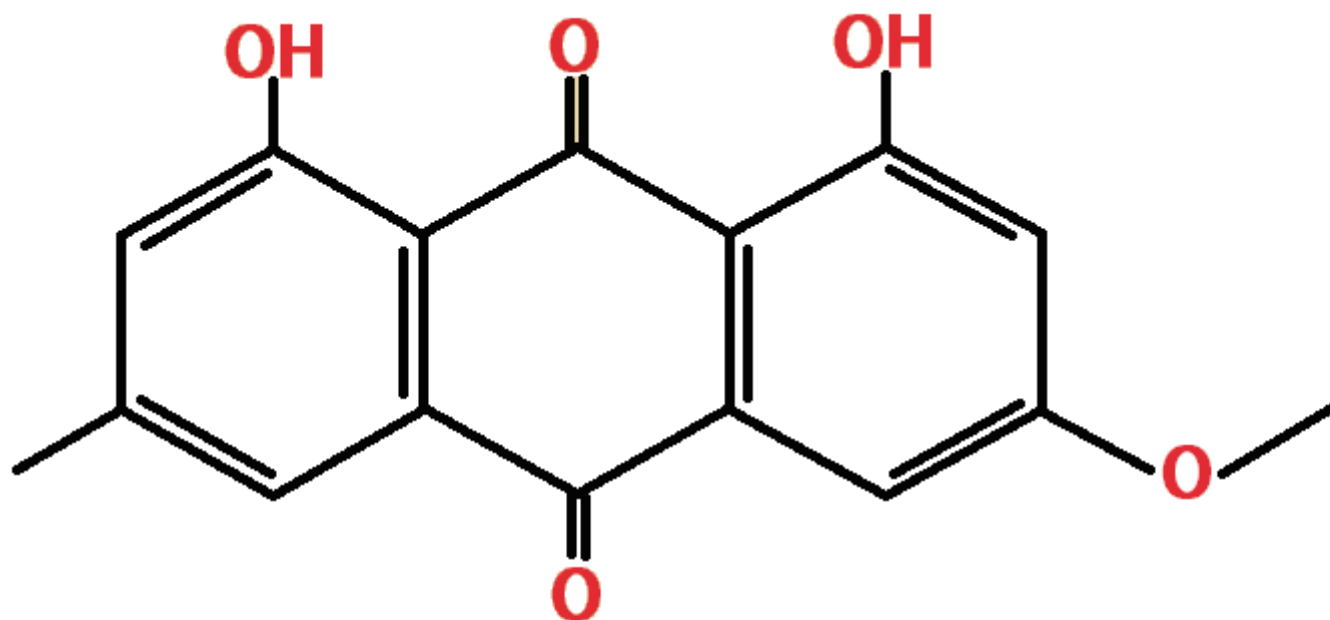

Supplement: Figure S3 [file peerj-04-1991-s003.pdf]
